# Editorial: Sexual and reproductive health among transgender and non-binary adolescents and young adults

**DOI:** 10.3389/frph.2024.1437349

**Published:** 2024-08-12

**Authors:** Nadia Dowshen, Diane Chen

**Affiliations:** ^1^ Department of Pediatrics, Children’s Hospital of Philadelphia, Philadelphia, PA, United States; ^2^ Department of Psychiatry and Behavioral Health, Ann & Robert H. Lurie Children’s Hospital of Chicago, IL, Chicago, United States

**Keywords:** transgender youth, non-binary youth, gender diverse adolescents, reproductive health, sexual health

**Editorial on the Research Topic**
Sexual and reproductive health among transgender and non-binary adolescents and young adults

The World Health Organization (WHO) defines sexual and reproductive health (SRH) as a state of physical, emotional, mental, and social well-being in all functions and processes related to sexuality and the reproductive system (1). While SRH is critical to general health, well-being, and quality of life for all people, more than 1% of adolescents and young adults (AYA) who identify as transgender and non-binary (TNB) have unique SRH needs and often lack access to culturally competent and age appropriate SRH care and education. This collection of articles spans the four key domains of SRH for TNB youth including (1) romantic/sexual relationships and experiences, and sexual function/satisfaction; (2) contraception and pregnancy prevention; (3) fertility and family building; and (4) HIV/STI prevention intervention.

Frequently SRH care and education for youth is focused on disease and pregnancy prevention and rarely considers the context of romantic relationships, sexual satisfaction and function which are key to normal adolescent development and health and wellbeing in adulthood. In this collection, Coyne et al. explore both the challenges and opportunities for online sexual exploration among a diverse sample of transfeminine youth. Data from United States (US)-based samples show that transfeminine individuals experience high levels of sexual assault and harassment making it particularly challenging for this population to find partners in a way that is safe and affirming (2). Many youth in the sample reported positive experiences with being able to explore relationships online without fear for their physical safety. However, many also discussed experiences of harassment and discrimination which were often compounded by racism for youth with intersecting minoritized racial identities. Perhaps most striking is that almost all of the youth reported never receiving any education or parental guidance about online dating or relationships suggesting an urgent need for implementing interventions for providers, parents and youth around these topics.

When TNB AYA engage in physical sexual relationships we want them to be prepared to do this in safe and healthy ways, ensuring knowledge and access to contraception and STI/HIV prevention. A newer tool for HIV prevention, Pre-Exposure Prophylaxis (PrEP), is a critical option for TNB youth who are placed at higher risk for HIV infection when compared to their cisgender counterparts, particularly Black, Indigenous, People of Color (BIPOC) and trans feminine youth who have the highest likelihood of HIV infection due to factors such as racism, sexism and transphobia (3). PrEP consists of taking pills daily, intermittently surrounding a sexual encounter, or an every eight-week injection. Unfortunately, awareness and uptake of PrEP is still very low among youth who need it the most. Rodriguez et al. similarly report that youth attending seven pediatric gender clinics in geographically diverse locations across the US that about one-third are unaware of PrEP, only 7% had ever been prescribed PrEP, and more than half had never had a conversation with a medical provider of PrEP. Additionally, many TNB AYA had low prep-related knowledge and many had erroneous concerns about PrEP's interaction with gender-affirming hormone therapy. Of note, the sample comprised only 7% Black and 25% transfeminine identified youth, signaling that youth who may need PrEP the most often face more barriers to gender-affirming care, which may be one of their only touch points with the healthcare system and critical opportunities to receive a prescription for PrEP. Further, given that discussions about PrEP are indicated for all sexually active adolescents we must better prepare clinicians providing gender-affirming care to both counsel and provide prescriptions for PrEP in this population of youth with high levels of indication.

In addition to supporting equity in SRH care and education for TNB AYA, we also must ensure providers and educational materials thoroughly and accurately address the effects of potential gender-affirming medical or surgical interventions on sexual health and wellbeing. Taylor et al. completed a content analysis of consent forms for gender-affirming hormone therapy and pubertal suppression to determine which SRH topics are discussed and how. The study found a wide range in the level of detail and tone with which four major topics were discussed: STIs, changes in function of sexual and reproductive organs, pregnancy and fertility, and cancer risk. While most consent forms indicated the possibility of infertility due to treatment, relatively few discussed the specifics of fertility preservation, with some consent forms encouraging preservation and some taking a negative or neutral tone. These findings underscore the need for standardization of consent forms and educational tools to be used to support youth and caregiver understanding of how treatment may impact SRH and what, if any, options are available to address these impacts.

Unfortunately, lack of access to high quality SRH care for TNBY is further compounded by the current political environment including legislation and other policies in more than twenty states in the United States to limit and ban gender-affirming medical care, with a particular focus on minors (4). Many of these laws and the debates surrounding them include misinformation about the impact of gender-affirming medical treatments on SRH including, for example, that puberty blockers and hormones “castrate” youth who receive them (5, 6). In fact, we know that youth who receive gender-affirming medical and/or surgical interventions have a number of options for fertility preservation and family building, even when interventions may temporarily or, in the case of surgery, permanently impair fertility. In the face of this type of widespread misinformation it is even more critical that youth-serving professionals and parents are prepared to provide high quality SRH information and services to TNB AYA. Future efforts are needed to ensure equity in access to SRH info and quality care for TNB AYA across the globe to ensure that they are able to grow up to be adults who have happy and healthy sexual lives and relationships as all people deserve.
